# Editorial: Innate Lymphoid Cells in Cancer: Friends or Foes?

**DOI:** 10.3389/fimmu.2021.804156

**Published:** 2021-11-18

**Authors:** Nicolas Jacquelot, Emilie Narni-Mancinelli, Alexander D. Barrow

**Affiliations:** ^1^ Princess Margaret Cancer Centre, University Health Network, Toronto, ON, Canada; ^2^ Aix Marseille Université, INSERM, CNRS, Centre d’Immunologie de Marseille-Luminy, Marseille, France; ^3^ Department of Microbiology and Immunology, The University of Melbourne and The Peter Doherty Institute for Infection and Immunity, Melbourne, VIC, Australia

**Keywords:** innate lymphoid cells, natural killer cells, immunotherapy, cancer, innate immunity

Innate lymphoid cells (ILCs) constitute the innate counterpart of T lymphocytes. They are devoid of antigen specific receptors and instead express a myriad of surface molecules allowing them to actively sense, and appropriately respond to, their tissue environment. This family of cells is classified into five subsets based on their developmental trajectories, the transcription factors and cytokines they express, and their effector functions. While type 1 ILCs (ILC1) only express the transcription factor T-bet, NK cells additionally express Eomes. Both subsets produce high levels of interferon (IFN)-γ and cytotoxic molecules, key features involved in the elimination of virus-infected and tumor cells. Type 2 ILCs (ILC2) express the transcription factors Gata3 and Rorα and secrete type 2 cytokines, such as IL-4, IL-5, IL-13 and GM-CSF, which are critical mediators of helminth expulsion and parasite elimination. Complementary to other ILC subsets, LTi cells and type 3 ILCs (ILC3) express the transcription factor Rorγt and produce the cytokines IL-17 and IL-22. LTi cells drive lymphoid organ development during embryogenesis whereas ILC3 contribute to intestinal homeostasis after birth through the promotion of epithelial cell growth and repair as well as the elimination of harmful extracellular pathogens.

ILCs can circulate around the body but predominantly reside within organs to maintain tissue homeostasis, potentially impacting the development and progression of every tumor type. In this regard, the review by An et al. extensively discusses the bidirectional immunomodulation of ILCs with the tumor microenvironment. This includes interactions with the extracellular matrix, other immune and non-immune cell types which express cytokines and growth factors that deeply influence the function of ILCs and their impact on cancer prognosis and treatment. In their review, they further highlight how targeting the heterogeneity and plasticity of the ILC family holds potential for cancer immunotherapy. Complementary to this work, Ducimetière et al., detail the bidirectional communication that exists between ILCs and the tumor microenvironment and discuss the impact of ILC migration *versus* local expansion in the tumor microenvironment in addition to the pathways and molecules involved in ILC interactions with innate and adaptive immunity, endothelial and tumor cells.

Numerous ILC tissue specificities have been reported. Remarkably, ILCs are enriched at mucosal sites, particularly in the intestinal tract, where they engage in activities essential for the preservation of epithelial barrier integrity. In this regard, Huang et al. highlight the dual role played by the different ILC subsets in the development, prognosis and treatment of colorectal cancer. ILC functions are dictated by their tissue environs, and it is now well appreciated that the tumor microenvironment largely differs according to tumor type. This tumor heterogeneity influences ILC activity, potentially driving divergent outcomes in cancer. This is particularly evident for ILC2 activation pathways in cancer. With this in mind, Ercolano et al. summarized our current knowledge of the role and impact of ILC2s in cancer, comprehensively detailing the pro- and anti-tumorigenic functions of this innate immune cell subset in hematological malignancies, urogenital and gastrointestinal cancers, melanoma, breast and lung tumors. Adding to this, Tumino et al. summarized the role of ILC1, ILC2 and ILC3 in human tumors. Similar to T cells, ILCs express many immune checkpoints at their membrane, further regulating their function. Tumino et al. reviewed the currently reported immune checkpoints expressed by ILCs in various settings including tumor development. Even though the contribution of ILCs in malignancy is only now beginning to emerge, these reviews highlight that, in addition to NK cells, ILCs clearly impact cancer development, progression, prognosis, and treatment.

Discovered more than 45 years ago, seminal studies in mice and humans have demonstrated that NK cells exhibit strong anti-tumor activities. NK cells mediate anti-tumor immune responses through the secretion of cytokines, chemokines and growth factors and the direct lysis of tumor cells. Their activity is dictated by a fine balance between activating and inhibitory receptors. Heightened engagement of these activating receptors promotes NK cell function and the release of effector molecules that kill target cells. In contrast, NK cells are equipped with inhibitory receptors which, when engaged, impair NK cell activity. For example, NK cells express the polymorphic Killer cell immunoglobulin-like receptors (KIR) that recognize HLA class I molecules, which are often downregulated by cancer cells. Consequently, these activating and inhibitory receptor family educate NK cells to recognize and kill target cells while sparing healthy cells. By analysing large cohorts of healthy individuals and leukemic patients from Southern China, Deng et al. observed major differences in their KIR haplotypes influencing NK cell cytotoxic function and patient prognosis. The genotyping of the *KIR A* haplotype has revealed higher homozygosity in healthy donors than in cancer patients. *KIR A* homozygosity was associated with increased NK cell cytotoxicity against leukemogenic cells having altered HLA expression, conferring increased protection against leukemia development.

NK cell anti-tumor function is also influenced by other components of the tumor microenvironment, including the extracellular matrix (ECM). Rossi et al. summarized in detail how the ECM and its components regulates NK cell activity. In particular, the ECM is enriched in TGF-β, known to promote the transdifferentiation of NK cells into ILC1-like cells. In non-small cell lung cancer patients, Verma et al. observed a downregulation of Eomes expression in circulating NK cells that is associated with disease progression. They recapitulated this phenotype in mice and further demonstrated that NK cells that have downregulated Eomes expression exhibit an ILC1-like phenotype with reduced anti-tumor effector function. Thus, in addition to activation and inhibitory receptors, there is a growing interest in this field to better understand the characteristics of the ECM and its impact on ILC function with the aim of designing new cancer therapies.

Malignant brain tumors are difficult to treat due to the unique anatomy of the brain, an immune privileged site that is poorly immunogenic. Consequently, tumors of the central nervous system often exhibit an immunosuppressive tumor immune microenvironment, representing a major therapeutic challenge to overcome. Sedgwick et al. review the emerging evidence for ILCs in brain tumor immunosurveillance, with a specific emphasis on NK cells. In their review, Sedgwick et al. further described various NK cell-based therapies currently under investigations in malignant brain tumors, potentially offering new therapeutic perspectives to patients. Patients with NK cell-enriched tumors often exhibit a more favourable prognosis. Moreover, different NK cell phenotypes may be associated with differential patient outcomes. Using NK cell transcriptional signatures and computational analyses of The Cancer Genome Atlas, Sun et al. demonstrated that low-grade glioma patient tumors enriched for a PDGF-DD activated NK cell phenotype correlates with a more favorable prognosis. PDGF-DD is a ligand for the activating receptor NKp44. Thus, PDGF-DD engagement of NKp44 may promote NK cell anti-tumor function and represent a clinically relevant pathway in low-grade glioma. In contrast to low-grade glioma, Sun et al. found that an IL-2 expanded NK cell phenotype was associated with a more favorable prognosis in bladder cancer. Together, these results indicate that NK cell signatures may be used as prognostic markers to further stratify patients in the clinic.

Collectively, this Research Topic covers the diverse roles and functions of ILC subsets in tumors and their impact on cancer patient prognosis and treatment. While the anti-tumor potential of NK cells in cancer is now well demonstrated, how other ILC subsets impact patient prognosis and treatment efficacy and whether it is feasible to harness their function in cancer remains, to date, largely unknown. This exciting new research field aims to better understand the role of ILCs in cancer and to harness the anti-tumor functions of these innate immune cells for the development of innovative next generation anti-cancer immunotherapies.

## Author Contributions

All authors listed, have made substantial, direct and intellectual contribution to the work, and approved it for publication.

## Conflict of Interest

The authors declare that the research was conducted in the absence of any commercial or financial relationships that could be construed as a potential conflict of interest.

## Publisher’s Note

All claims expressed in this article are solely those of the authors and do not necessarily represent those of their affiliated organizations, or those of the publisher, the editors and the reviewers. Any product that may be evaluated in this article, or claim that may be made by its manufacturer, is not guaranteed or endorsed by the publisher.

